# Dynamically regulated miRNA-mRNA networks revealed by exercise

**DOI:** 10.1186/1472-6793-13-9

**Published:** 2013-06-07

**Authors:** Alexander G Tonevitsky, Diana V Maltseva, Asghar Abbasi, Timur R Samatov, Dmitry A Sakharov, Maxim U Shkurnikov, Alexey E Lebedev, Vladimir V Galatenko, Anatoly I Grigoriev, Hinnak Northoff

**Affiliations:** 1 Moscow State University, Leninskie Gory, Moscow 119991, Russia; 2 The Institute of General Pathology and Pathophysiology, Russian Academy of Medical Sciences, Baltiiskaya str. 8, Moscow 125315, Russia; 3 SRC Bioclinicum, Ugreshskaya str 2/85, Moscow 115088, Russia; 4 Institute of Clinical and Experimental Transfusion Medicine (IKET), University of Tübingen, Otfried-Müller-str. 4/1, Tübingen 72076, Germany; 5 Institute for Biomedical Problems, Russian Academy of Sciences, Khoroshevskoe road 76a, Moscow 123007, Russia

**Keywords:** Exercise, Regulation, miRNA-mRNA networks

## Abstract

**Background:**

MiRNAs are essential mediators of many biological processes. The aim of this study was to investigate the dynamics of miRNA-mRNA regulatory networks during exercise and the subsequent recovery period.

**Results:**

Here we monitored the transcriptome changes using microarray analysis of the whole blood of eight highly trained athletes before and after 30 min of moderate exercise followed by 30 min and 60 min of recovery period. We combined expression profiling and bioinformatics and analysed metabolic pathways enriched with differentially expressed mRNAs and mRNAs which are known to be validated targets of differentially expressed miRNAs. Finally we revealed four dynamically regulated networks comprising differentially expressed miRNAs and their known target mRNAs with anti-correlated expression profiles over time. The data suggest that hsa-miR-21-5p regulated TGFBR3, PDGFD and PPM1L mRNAs. Hsa-miR-24-2-5p was likely to be responsible for *MYC* and *KCNJ2* genes and hsa-miR-27a-5p for *ST3GAL6*. The targets of hsa-miR-181a-5p included ROPN1L and SLC37A3. All these mRNAs are involved in processes highly relevant to exercise response, including immune function, apoptosis, membrane traffic of proteins and transcription regulation.

**Conclusions:**

We have identified metabolic pathways involved in response to exercise and revealed four miRNA-mRNA networks dynamically regulated following exercise. This work is the first study to monitor miRNAs and mRNAs in parallel into the recovery period. The results provide a novel insight into the regulatory role of miRNAs in stress adaptation.

## Background

MiRNAs are one family of small (20–22 nucleotides) non-coding RNAs. They regulate gene expression post-transcriptionally through binding to the complementary sites of target mRNAs in the 3′-UTR, and play an important role in regulating diverse biological processes [1].

Recently, miRNA have been demonstrated as regulators of processes involved in physiological stress adaptation, including inflammation [2], angiogenesis [3], mitochondrial metabolism [4], muscle force generation [5]. However, just a few studies were published to date describing the changes in miRNA expression during exercise of different intensity [6-12]. They did not include the analysis of post-exercise recovery period and thus provided no information concerning dynamics of the predicted miRNA-mRNA regulatory pairs. Detailed investigation of miRNA-mRNA networks specifically regulated by exercise could reveal important biomarkers of exercise physiology and would provide for deep insight into the molecular control of the stress response.

MiRNAs regulate target gene expression in different ways including mRNA degradation and translation inhibition [1]. The target genes which were regulated by miRNAs through mRNA degradation are anti-correlated with the miRNA regulators. In this study, for the first time whole transcriptome changes were monitored during exercise followed by 30 min and 60 min of recovery period and differentially expressed mRNAs and miRNAs were analysed resulting in identification of four dynamically regulated miRNA-mRNA networks.

## Results and discussion

### Anthropometric and physiological data

To exclude possible effects of gender, only male subjects were recruited for this study. Anthropometric and physiological parameters of athletes are presented in Table 1. Before exercise the serum lactate level was 1.7 ± 0.4 mM. After exercise, it was mildly elevated, but still below 4.0 mM, confirming that the exercise performed was moderate without transgression of the anaerobic threshold.

**Table 1:** Anthropometric and physiological data

**Parameter**	**Value**
Age (year)	21.7 ± 2.6
Body mass (kg)	74.9 ± 2.3
Height (cm)	185.3 ± 3.5
Body mass index (kg/m^2^)	21.8 ± 1.2
Heart rate before exercise(bpm)	57.3 ± 1.9
Heart rate after exercise (bpm)	179.4 ± 3.2
Blood pressure before exercise ( mmHg)	120/66 ± 3/2
VO_2max_ (ml min^-1^ kg^-1^)	74.8 ± 3.3

Values are mean ± SD.

Branched-chain amino acids (BCAA) include three structurally related amino acids Leucine (Leu), Isoleucine (Ile), and Valine (Val). The initial steps of their degradation are catalyzed by the same set of mitochondrial enzymes, and therefore, the BCAA behave as a very homogenous group. Their regulation is performed by short-term metabolic control reflecting consuming of energy sources. It has been shown previously that an acute bout of prolonged exercise increases the rate of BCAA oxidation by skeletal muscle [13]. We observed a slight increase in the BCAA level immediately after exercise followed by a decline below base level during recovery (Table 2). The ratio of citrulline (Cit) to ornithine (Orn) is indicative of the ornithine carbamoylphosphate transferase activity and characterizes the regulation of the urea cycle pathway [14]. This ratio had a tendency to increase (Table 2). The ratios of methionine sulfoxide (Met-SO) to methionine (Met) and tyrosine (Tyr) to phenylalanine (Phe) indicate oxidative stress [15,16]. We found a decrease in Met-SO/Met and a slight increase in Tyr/Phe (Table 2).

**Table 2:** Aminoacids before and in response to exercise

	**BCAA, μM**	**Cit/Orn**	**Met-SO/Met**	**Tyr/Phe**
Pre	567.63 ± 172.16	0.35 ± 0.09	2.28 ± 1.73	0.83 ± 0.15
Post (E)	612.83 ± 99.68	0.34 ± 0.11	0.92 ± 0.21	0.91 ± 0.18
Rest 30 min (R1)	496.89 ± 128.31	0.42 ± 0.09	1.71 ± 0.92	0.91 ± 0.19
Rest 60 min (R2)	417.75 ± 58.2	0.5 ± 0.15	1.21 ± 0.47	0.9 ± 0.16

Abbreviations: *BCAA* Branched-Chain Amino Acids, *Cit* Citrulline, *Orn* ornithine, *Met-SO* Methionine Sulfoxide, *Met* Methionine, *Tyr* Tyrosine, *Phe* Phenylalanine.

The data summarized in Table 2 confirmed that the exercise was moderate and athletes reacted as normal healthy subjects [17].

### Flow cytometry analysis

Changes in white blood cell subpopulations in response to exercise are presented in Table 3. Total white blood cell counts revealed the expected exercise-induced leukocytosis. NK lymphocytes (defined as CD3–, CD16/56+) substantially contributed to the observed changes which was consistent with the published data [18]. However NK-specific mRNAs (e.g., coding for KIR receptors) in the whole blood did not demonstrate similar nearly 3-fold increase (see Additional file 1) thus confirming that our subsequent microarray analysis showed true changes in RNA expression.

**Table 3:** Changes in white blood cell subpopulations in response to an exercise

	**Pre**		**Post (E)**		**Fold change relative**
	**Absolute, cells/ul**	**Relative, %**	**Absolute, cells/ul**	**Relative, %**	
WBC	5710 ± 1170		7705 ± 2000		
LY	2387 ± 674	41.8 ± 11.8	3989 ± 932	69.9 ± 12.1	1.67
СD3+	1616 ± 549	28.3 ± 9.6	2349 ± 901	41.1 ± 11.7	1.45
СD3 + CD4+	1020 ± 413	17.9 ± 7.2	1189 ± 419	20.8 ± 5.4	1.17
СD3 + CD8+	596 ± 265	10.4 ± 4.6	1124 ± 532	19.7 ± 6.9	1.89
СD3-CD16+	494 ± 203	8.7 ± 3.6	1380 ± 523	24.2 ± 6.8	2.79
СD3-CD56+	393 ± 217	6.9 ± 3.8	1111 ± 468	19.5 ± 6.1	2.83
CD19+	227 ± 124	4.0 ± 2.2	308 ± 221	5.4 ± 2.9	1.36

Values are mean ± SD.

### miRNA and mRNA differential expression profiles

The miRNA and mRNA expression profiles in the whole blood for each time point and each athlete were determined using microarray analysis. PAXgene RNA tubes enable the isolation of intracellular RNA of circulating leukocytes, including B cells, T cells, neutrophils, monocytes, and other less abundant cell types. Furthermore, a large proportion of reticulocyte-derived globin mRNA is prepared from PAXgene blood RNA tubes, as it has been demonstrated previously [19]. Affymetrix GeneChip Human Gene 1.0 ST arrays contain both miRNA and mRNA probes and are capable of measuring about 20,000 mRNAs and 200 miRNAs.

**Figure 1:**
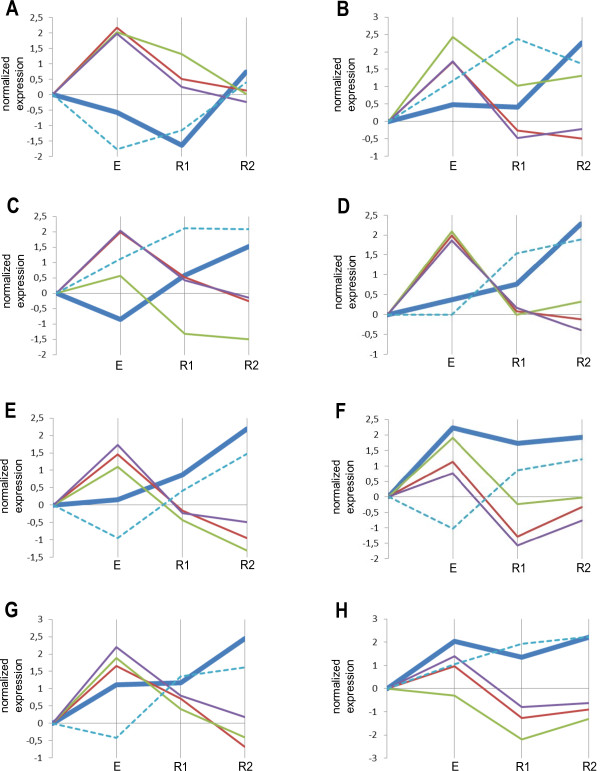
**Expression profiles of hsa-miR-21-5p and its target mRNAs.** (**A**-**H**) the tested athletes. **E**, after 30 min of exercise; R1, after 30 min relaxation; R2, after 60 min relaxation. Bold blue, hsa-miR-21-5p; brown, TGFBR3; green, PDGFD; violet, PPM1L; light blue, RHOBTB3. Solid lines indicate validated mRNA targets and dashed line indicates predicted potential mRNA target.

**Figure 2:**
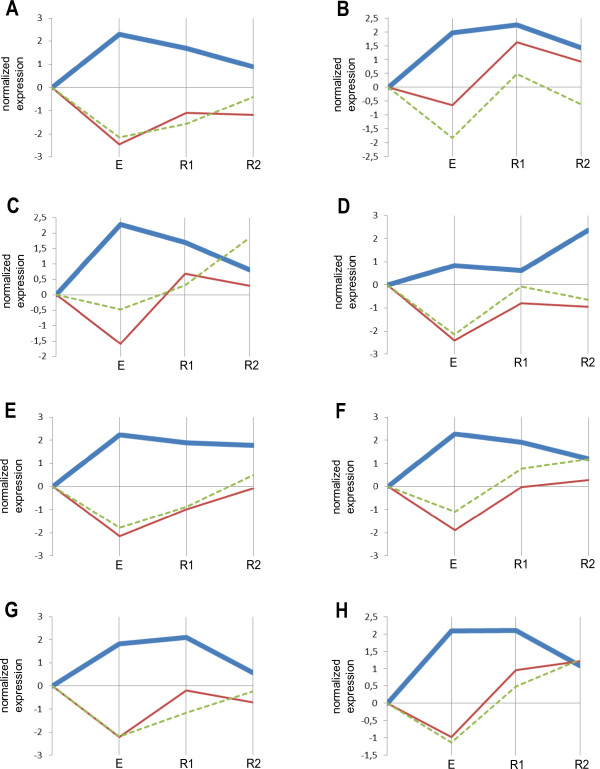
**Expression profiles of hsa-miR-24-2-5p and its target mRNAs.** (**A**-**H**) the tested athletes. **E**, after 30 min of exercise; R1, after 30 min relaxation; R2, after 60 min relaxation. Bold blue, hsa-miR-24-2-5p; brown, MYC; green, KCNJ2. Solid line indicates validated mRNA target and dashed line indicates predicted potential mRNA target.

**Figure 3:**
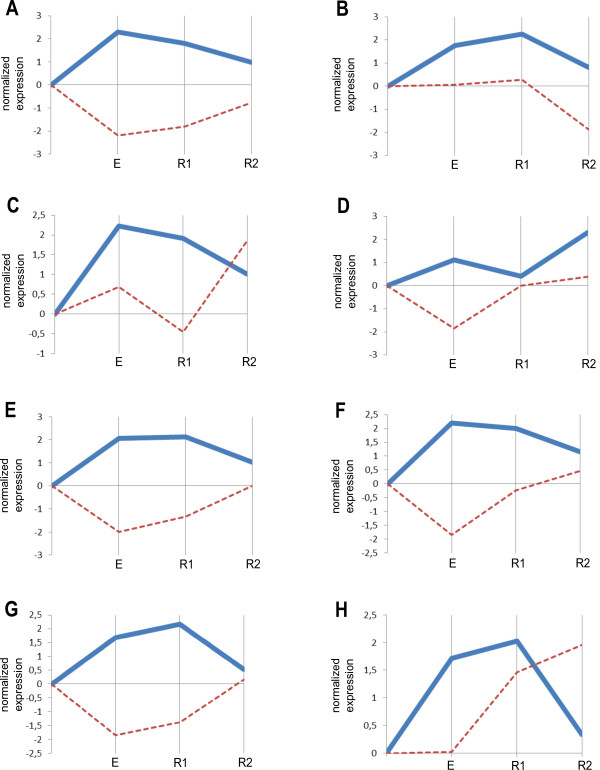
**Expression profiles of hsa-miR-27a-5p and its target mRNA.** (**A**-**H**) the tested athletes. **E**, after 30 min of exercise; R1, after 30 min relaxation; R2, after 60 min relaxation. Bold blue, hsa-miR-27a-5p; dashed brown, predicted potential target mRNA ST3GAL6.

**Figure 4:**
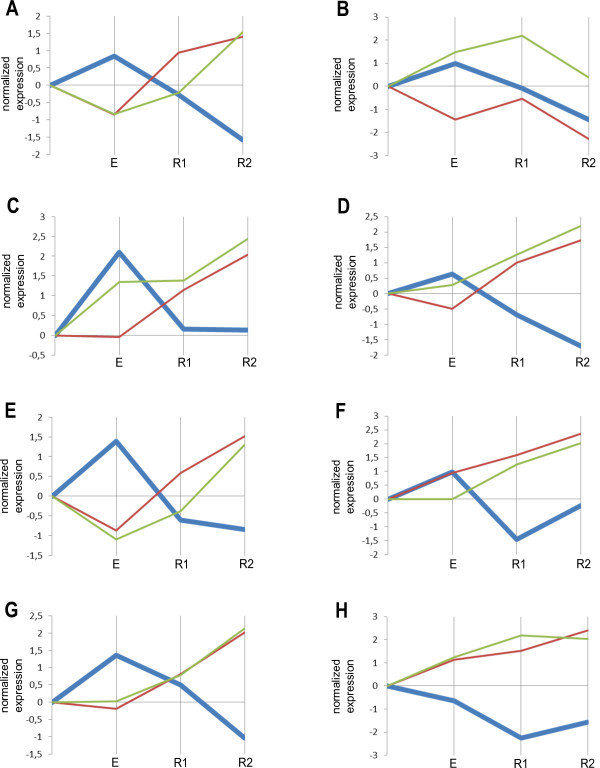
**Expression profiles of hsa-miR-181a-5p and its validated target mRNAs.** (**A**-**H**) the tested athletes. **E**, after 30 min of exercise; R1, after 30 min relaxation; R2, after 60 min relaxation. Bold blue, hsa-miR-181a-5p; brown, ROPN1L; green, SCL37A3.

The data are presented in Additional file 1. 298 mRNAs and 5 miRNAs were changed significantly (at least 40% change with the adjusted P-value threshold of 0.05), including hsa-miR-21-5p, hsa-miR-24-2-5p, hsa-miR-27a-5p, hsa-miR-181a-5p and hsa-miR-181b-5p. Remarkably, hsa-miR-24-2-5p is clustered with hsa-miR-27a-5p, and hsa-miR-181a-5p is clustered with hsa-miR-181b-5p. Consistently, the clustered miRNAs exhibited similar expression profiles over time (see Additional file 1 and Figures 1, 2, 3 and 4).

### Pathway analysis of differentially expressed mRNAs

All 298 differentially expressed mRNAs were analysed for enriched metabolic pathways. Table 4 indicates the revealed pathways including immune response and glycoproteins. As expected, they were previously reported to be associated with exercise [6,8] thus confirming the relevance of our experimental model.

**Table 4:** Pathway analysis of differentially expressed mRNAs

**Pathway**	**Number of genes**	**Adjusted P-value**
Glycoprotein	76	3.00E-09
Natural killer cell mediated cytotoxicity	22	1.80E-15
Immune response	13	3.10E-03
Response to wounding	7	8.70E-01
Inflammatory response	7	1.90E-01
Regulation of lymphocyte mediated immunity	5	1.40E-01
Leukocyte activation	5	8.90E-01
Response to mechanical stimulus	3	3.80E-02
Stress response	3	5.90E-02
Cytolysis	3	3.20E-02

### Pathway analysis of mRNA targets of differentially expressed miRNAs

All 5 differentially expressed miRNAs have 1136 validated target mRNAs in total. We performed pathway enrichment analysis for these mRNAs (Table 5). Again, the revealed pathways are highly relevant to exercise, e.g. transcription regulation, apoptosis, response to stress etc.

**Table 5:** Pathway analysis of all validated mRNA targets of differentially expressed miRNAs

**Pathway**	**Number of genes**	**Adjusted P-value**
Transcription regulation	283	2.03E-13
Apoptotic process	157	2.59E-11
Cell cycle	148	5.95E-12
Regulation of kinase activity	76	1.08E-08
Regulation of cellular response to stress	34	1.63E-04
p53 signaling pathway	19	4.26E-08
E2F transcription factor network	19	1.51E-07
Validated targets of C-MYC transcriptional activation	14	1.08E-03

**Figure 5:**
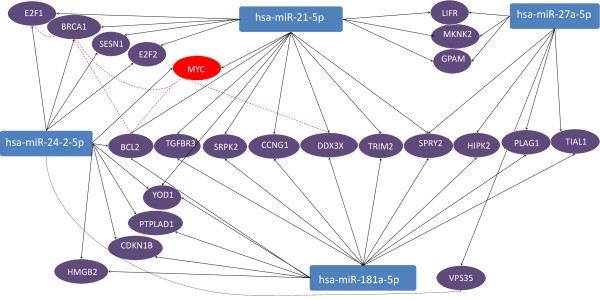
**Regulatory network of mRNAs validated to be the targets for 2 or 3 differentially expressed miRNAs.** All mRNAs belong to three enriched pathways listed in Table 6. Red dashed lines connect mRNAs of proteins reported to functionally interact with each other. MYC is highlighted with red oval indicating that this mRNA was further identified to be a component of dynamically regulated miRNA-mRNA network.

It has been demonstrated that the same mRNAs can be targeted by more than one miRNA which provides for more efficient and specific regulation [20,21]. We found 49 mRNAs which are known to be validated targets for 2 or even 3 differentially expressed miRNAs. They have higher potential to be involved in exercise-induced regulation. Table 6 shows the pathways enriched with some of these mRNAs. Notably, these exercise-relevant pathways (cell death, stress response, proliferation) comprise significant number of intersecting mRNAs. Figure 5 presents the identified regulatory miRNA-mRNA network for all 3 revealed pathways. Interestingly, some of these genes are known to interact with each other. Namely transcription factor MYC was reported to be functionally associated with RNA helicase DDX3X [22], apoptosis regulator BCL2 [23] and tumor suppressor BRCA1 [24]. BRCA1 in turn interacts itself with BCL2 [25] and transcription factor E2F1 [26]. The presented data support the regulatory role of identified miRNAs in response to exercise.

**Table 6:** Pathway analysis of mRNAs validated to be targets for 2 or 3 differentially expressed miRNAs

**Pathway**	**Number of genes**	**Adjusted P-value**
Cell death	17	7.64E-07
Regulation of cell proliferation	15	2.10E-07
Cellular response to stress	12	5.35E-05

### Dynamically regulated miRNA-mRNA networks

We monitored the transcriptome expression level before and following exercise and this allowed us to reveal dynamically regulated miRNA-mRNA networks. We used a two-step approach to identify the mRNA targets for the differentially expressed miRNAs. First, we looked for anti-correlating groups of miRNAs and mRNAs expression of which over time tended to have opposite profiles. Target mRNA degradation is one of the mechanisms of miRNA action when their perfect complementarity occurs [1,27]. Thus, the second step of our strategy was either analysis of published data and selection of experimentally validated target mRNAs for a given miRNA or theoretical prediction of miRNA targets based on their complementarity, using one of the most popular web resources TargetScan [28]. The expression profiles and biological function of selected miRNAs and mRNAs were analysed in more detail. Based on this analysis, our final identified miRNA-mRNA pairs have a high probability of being involved in the regulation of exercise-related physiological processes.

### hsa-miR-21-5p

MiRNA hsa-miR-21-5p demonstrated different expression profiles over time (adjusted P-value 0.0039) however remarkably anti-correlating with experimentally validated target mRNAs TGFBR3 (adjusted P-value 1.079E-06), PDGFD (adjusted P-value 1.28E-06) and PPM1L (adjusted P-value 6.78E-07) (Figure 1). RHOBTB3 mRNA (adjusted P-value 0.002) predicted by TargetScan to be a potential target for hsa-miR-21-2-5p behaved similarly. We observed the up-regulation of hsa-miR-21-5p one hour after exercise. The differences in kinetics can be potentially explained by individualities of each athlete.

The expression level of hsa-miR-21-5p itself is known to be stress-responsive and play an important role in heart failure [29] and renal ischemia reperfusion injury [30]. Notably the up-regulation of circulating hsa-miR-21-5p was recently reported to occur in plasma upon exercise [6]. The overall action of hsa-miR-21-5p has been described by several authors to be strongly anti-inflammatory [6,31]. Note, that 60 min into relaxation, there was an up-regulation of hsa-miR-21-5p in all 8 subjects. This may reflect the self-protective anti-inflammatory reaction to exercise.

We identified four pairing targets for this miRNA namely TGFBR3, PDGFD, PPM1L, and RHOBTB3. TGFBR3 is a transforming growth factor (TGF)-beta type III receptor, mRNA of which is known to be up-regulated in the peripheral blood leukocytes in allograft rejection-prone recipients after intestinal transplantation thus mediating innate and adaptive inflammatory functions of leukocytes [32]. PDGFD encodes for platelet derived growth factor D [33], a member of the platelet-derived growth factor family which can regulate many cellular processes, including cell proliferation, apoptosis, transformation, migration, invasion, angiogenesis and metastasis [34]. PPM1L encodes a protein phosphatase gene [35] responsible for the regulation of stress-activated protein kinase signaling cascade and apoptosis [36]. Finally, the fourth identified target for hsa-miR-21-5p is RHOBTB3 mRNA [33] which encodes for the Rho GTPase regulating the membrane traffic of proteins [37]. Interestingly, this mRNA proved to be a blood biomarker of psychosis and shows a decreased expression level in high hallucinations states [38].

### hsa-miR-24-2-5p

MiRNA hsa-miR-24-2-5p (adjusted P-value 0.00017) was up-regulated immediately after exercise, then tended to decrease during the recovery period except athlete D (Figure 2). In our study for the first time we report the reaction to exercise of this miRNA which is known to have protective effects on myocytes against myocardial ischaemia/reperfusion-induced apoptosis [39].

MYC mRNA (adjusted P-value 0.00013) which is known to be the target for this miRNA from the literature, and KCNJ2 mRNA (adjusted P-value 0.00023) predicted by TargetScan to be a potential target essentially followed anti-profile of hsa-miR-24-2-5p. The protein encoded by the *MYC* gene is a multifunctional, nuclear phosphoprotein that plays a role in cell cycle progression, apoptosis and cellular transformation. It functions as a transcription factor that regulates transcription of specific target genes. Interestingly, hsa-miR-24-2-5p is known to be up-regulated during hematopoietic cell terminal differentiation suppressing MYC expression [40]. Thus the increase of this miRNA we observed (Figure 2) may reflect the regulation of hematopoiesis upon exercise. Remarkably, MYC mRNA is among those which are regulated by 2 differentially expressed miRNAs, namely hsa-miR-21-5p and hsa-miR-24-2-5p (Figure 5), however its expression profile is anti-correlated with only the profile of hsa-miR-24-2-5p (Figures 1 and 2).

KCNJ2 protein is an integral membrane protein and inward-rectifier type potassium channel participating in establishing the action potential waveform and excitability of neuronal and muscle tissues [41]. This mRNA expressed in peripheral blood lymphocytes is a biomarker for Parkinson’s disease [42].

### hsa-miR-27a-5p

MiRNA hsa-miR-27a-5p is clustered with hsa-miR-24-2-5p and behaved similarly to it increasing after exercise and decreasing during the recovery period except athlete D (Figure 3) with the adjusted P-value 0.00012. This miRNA was reported to promote myoblast proliferation by reducing the expression of myostatin [43].

The only mRNA target identified is predicted by TargetScan ST3GAL6. The encoded protein belongs to the sialyltransferase family and is responsible for the synthesis of selectin ligands [44].

### hsa-miR-181a-5p

hsa-miR-181a-5p tended to increase after exercise and then to down-regulate during the first as well as the second period of the relaxation time (Figure 4) with an adjusted P-value of 5.83E-05. The observed differential expression of hsa-miR-181a-5p in our athletes is consistent with previously published results [7,8]. This miRNA is characterized as a regulator of hematopoietic lineage differentiation [45] and a modulator of T cell sensitivity and selection [46]. Radom-Aizik showed up-regulation of this miRNA after 30 min interval exercise. They related it to increased T cell responsiveness and reduced susceptibility to infection due to physical activity. In our study 7 subjects showed up-regulation immediately after exercise (Figure 4).

The mRNAs ROPN1L (adjusted P-value 0.00024) and SLC37A3 (adjusted P-value 0.0019) were previously validated to be targets for hsa-miR-181a-5p and demonstrated pronounced anti-correlation with the miRNA expression profile. The *ROPN1L* gene encodes a member of the ropporin family. The encoded protein is involved in the targeting towards specific physiological substrates of Protein Kinase A, regulating glycogen, sugar, and lipid metabolism [47]. The SLC37A3 protein belongs to transmembrane sugar transporters and is responsible for sugar metabolism [48].

## Conclusion

We have identified metabolic pathways enriched with differentially expressed mRNAs and with mRNA targets of differentially expressed miRNAs, including mRNAs known to be regulated by 2 or 3 miRNAs described here. The result supports previously published data. Moreover, we revealed four miRNA-mRNA networks dynamically regulated following exercise. These observations provide a novel insight into the potential regulatory role of miRNAs in the numerous physiological processes involved in stress adaptation.

## Methods

### Ethical approval and study participants

Eight national level ski athletes took part in this study. None of them suffered from acute or chronic diseases or reported intake of medication. Participants were informed about the nature, purpose, and potential risks of the experiments and signed an informed consent statement approved by the ethics committee of Scientific Research Center Bioclinicum (Moscow, Russia).

### Anthropometric measurements

Height, weight, medical historical data and resting vital signs were recorded at the time of enrolment.

### Exercise test protocol

In order to determine the VO_2max_ values, each subject performed a treadmill test with an incremental step protocol until exhaustion as described previously [47]. VO_2max_ was calculated as described [49]. The anaerobic threshold was calculated using the standard V-slope method [50].

Two weeks later, athletes participated in the main exercise, consisting of running at 80% VO_2_ peak for 30 min on a treadmill. The exercise was performed during the morning hours (between 8 and 11 a.m.) keeping the exact test time for each participant constant.

### Lactate concentration

Lactate concentration in capillary blood was measured electrochemically using the automated analyzer Biosen C_Line (EFK Diagnostic, Germany).

### Analysis of aminoacids

Sera derived from the venous blood samples were analyzed for aminoacids in Genome Analysis Centre (Helmholtz Zentrum, Munich, Germany) using the BIOCRATES AbsoluteIDQ p150 Kit (Biocrates Life Sciences AG, Innsbruck, Austria) in a combined FIA-MS/MS and LC-MS/MS assay as recommended by the manufacturer and described previously [51].

### Blood sampling

Venous blood was collected at four time points during the exercise. A 20-gauge intravenous catheter was placed antiseptically into a dorsal hand vein or a vein in the distal forearm as dictated by favourable anatomy using the Seldinger technique and then secured with tape. During ME 2.5 ml of blood was collected in PAXgene blood RNA tube, 7 ml in a Serum separator tube (BD, USA) and 4.5 ml in a tube containing buffered tri-sodium citrate (BD, USA) for flow cytometry analysis at baseline (prior to exercise testing) and immediately post-exercise (within 1 min of completion of exercise testing). After 30 min of rest and after 60 min of rest following exercise testing 2.5 ml of blood was collected in PAXgene blood RNA tubes.

### RNA extraction

According to the Affymetrix Manual P/N 701880 Rev. 4 total RNA was extracted using the PAXgene Blood RNA kit as recommended by the manufacturer. RNA concentrations were determined by the Nanodrop photometer (NanoDrop, USA). RNA quality was checked using the Agilent Bioanalyser 2100 System (Agilent Technologies, USA). For all samples RNA integrity number (RIN) was greater than 7.

### Flow cytometry analysis

Flow cytometry analysis of blood samples was performed using fluorescently labeled antibodies against B and T cell receptors and natural killer (NK) cell markers. Cells were labeled with antibodies against CD3 (FITC), CD4 (PE), CD8 (PE), CD16 (FITC), CD56 (PE), CD19 (FITC) (Sorbent, Russia), where NK cells were distinguished from the rest of the lymphocytes via positive expression of CD56 and negative expression of CD3.

The samples were analyzed on a FACScan Calibur flow cytometer (BD Biosciences, USA) and leukocytes were gated based on forward and side scatter properties. Events in the range of 40,000–200,000 were collected depending on the occurrence of the investigated leukocyte population, and analyzed with CELLQuest Pro analysis software (BD Biosciences, USA). To ensure flow cytometric standardization, the voltage settings were updated daily using ‘Calibrate Beads’ (BD Biosciences, USA).

### Microarray analysis

RNA samples were prepared according to manufacturer’s instructions (Affymetrix Manual P/N 701880 Rev. 4) as described elsewhere [52]. The samples were hybridized on GeneChip Human Gene 1.0 ST Arrays containing both miRNA and mRNA probes (Affymetrix, USA) for 16 h at 45°C. Arrays were washed to remove non-specifically bound nucleic acids and stained on Fluidics Station 450 (Affymetrix) using FS450_0007 protocol followed by scanning on a GeneChip Scanner 3000 7G system (Affymetrix). The microarray CEL files have been deposited in the GEO database (accession GSE46075).

### Microarray data processing

Microarray data was processed using bioconductor [53] xps package implementation of RMA [54]. At the first step background correction was performed based on a global model for the distribution of probe intensities [54]. Then a quantile normalization algorithm [55] (so-called probe-level normalization) was applied to the preprocessed data. Finally, fitting a robust linear model using Tukey’s median polish procedure [56] was done to convert probe intensities to the expression levels of probesets.

The statistical analysis of microarray data was performed using bioconductor [53] package limma [57]. The analysis was based on a generalized linear model [57,58] approach. In this approach one constructs a linear data model with a structure determined by the experiment layout, and then fits this model to the actual data. The linear model is defined in terms of a so-called design matrix. The number of rows in this matrix coincides with the number of experiment samples, and the number of columns coincides with the number of factors that have an essential influence on the measurable values. The value at the i-th row and j-th column of a design matrix specifies an effect of a factor j on a sample i. Each measurable value (i.e., each probeset) in this approach is analysed independently. For each probeset a vector of its expression values E is represented in the form E = Dβ + ϵ, where D is a design matrix, β is a vector of coefficients indicating values of each factor’s actual influence on the analyzed probeset, ϵ is a vector of error, and model fitting consists in the minimization of “error term” ϵ by finding optimal coefficients β. After the coefficients β are computed for each probeset, one can test various hypotheses on the structure of considered factors. For example, in order to find probesets that are affected by a factor i, one should search for the probesets with βi statistically different from zero.

The general linear model was applied in the analysis of the studied transcriptome changes as follows. The model that takes into account both experiment time points and athletes individual features was used. Thus, the total number of factors was eleven: c1, …, c8 correspond to an expression level of each athlete in a normal state (for all samples the effect of these factors is set to 1 if a sample and a factor correspond to the same athlete, and set to 0 otherwise), and d1, d2, d3 correspond to the changes induces by the exercises, exercises and 30-minutes relaxation, and exercises and 60-minutes relaxation respectively (for all samples the effect of these factors is set to 1 if a sample and a factor correspond to the same experiment time point, and set to 0 otherwise). The total number of samples was 32: 4 samples for each athlete.

For each pair of experiment time points the detection of probesets with reliable difference between time points was performed. The probeset was considered to have a reliable difference between time points k, m if an adjusted p-value of an equality βk = βm (where βl was an actual influence of exercises at experiment time point for l = 1,2,3, and β0 = 0) was less than 0.05, and a log-fold change was greater than 0.484 (this threshold corresponds to an intensity change by more than 40%). The Benjamini-Hochberg [59] algorithm was used for multiple testing adjustment. The minimum adjusted p-value for all pairs of time points is indicated for each differentially expressed probeset in the Table of differentially expressed mRNAs and miRNAs (Additional file 1) as a statistical significance value.

### Pathway analysis

Bioinformatic analysis was performed using DAVID online tool (http://david.abcc.ncifcrf.gov) as described elsewhere [60]. So all the analyzed genes were classified into several functional groups, and the groups that may be potentially associated with physiological stress were considered and listed in the tabs of excel document. P-values on the tabs are modified Fisher Exact P-Values. When members of two independent groups can fall into one of two mutually exclusive categories, Fisher Exact test is used to determine whether the proportions of those falling into each category differs by group. In DAVID annotation system, Fisher Exact test is adopted to measure the gene-enrichment in annotation terms.

## Abbreviations

miRNA: microRNA; mRNA: messenger RNA.

## Competing interests

The authors declare that they have no competing interests.

## Authors’ contributions

Conception and design of the experiments: AGT, AIG. Collection, analysis and interpretation of data: DVM, DAS, MUS, AEL, VVG. Drafting the article and revising it critically TRS, AGT, AA, HN. All authors read and approved the final manuscript.

## Supplementary Material

Additional file 1 Differentially expressed mRNAs and miRNAs. Click here for file
